# MRI-morphometric characterization of Chiari malformation types 0 and 1 with syringomyelia: implications for diagnosis and pathogenesis

**DOI:** 10.1007/s10072-026-08820-z

**Published:** 2026-03-13

**Authors:** Enver I. Bogdanov, Aisylu T. Faizutdinova, John D. Heiss

**Affiliations:** 1 Department of Neurology, Kazan State Medical University, Butlerov str. 49, Kazan, 420012 Russian Federation; 2 Surgical Neurology Branch, National Institute of Neurological Disorders and Stroke, National Institutes of Health, 10 Center Drive, 10-3D20, MSC-1414, Bethesda, MD MD 20892-1414 United States of America

**Keywords:** Chiari malformation type 1, Chiari malformation type 0, Syringomyelia, Small posterior cranial fossa, Foramen magnum

## Abstract

**Background:**

Primary Chiari malformation type 1 (CM1) and type 0 (CM0) appear to share features of overcrowding of the posterior cranial fossa (PCF) neural elements. We carried out the present study to identify anatomical features differentiating primary Chiari malformation type 1 (CM1) from Chiari malformation type 0 (CM0) and normal subjects, and then used those anatomic features for CM1 and CM0 phenotyping and diagnosis.

**Methods:**

This study included two groups of adult patients: CM0 with syringomyelia (SM) (*CM0-SM*) having cerebellar tonsillar descent ≤ 2 mm below the foramen magnum (FM), CM1 with syringomyelia (*CM1-SM)* with tonsillar herniation (TH) > 3 mm, and normal controls. Clinical data and MRI parameters were analyzed.

**Results:**

CM1 and CM0 associated with SM share anatomic characteristics of PCF hypoplasia, size reduction, and flattening, and share clinical findings of occipital headache and cervical central myelopathy. CM1 has more pronounced PCF hypoplasia, narrower FM CSF pathways, and more expansive and extensive syringes than CM0, while CM0 has reduced FM size. Combining two MRI morphometric markers predicted CM1 with a sensitivity of 94%, and three markers predicted CM0 with a sensitivity of 90%. In CM1 and CM0, the descent of the PCF neural structures, assessed by TH and obex descent relative to the FM, correlated with PCF flattening and FM size but not with PCF size.

**Conclusion:**

Four PCF morphometric markers independent of TH can accurately predict the presence of CM0 and CM1 associated with syringomyelia.

**Supplementary Information:**

The online version contains supplementary material available at 10.1007/s10072-026-08820-z.

## Introduction

The diagnosis of primary Chiari I malformation (CM1) typically requires one or both cerebellar tonsils to be at least 5 mm below the foramen magnum (FM) on a midsagittal, T1-weighted MRI image. Interestingly, 9% of CM1 surgical series and 33% of neurology department patients with CM1-like symptoms had tonsillar herniation (TH) of less than 5 mm [1, 2]. The severity of symptoms does not always correlate with the degree of TH, and many experts believe that TH under 5 mm does not exclude a CM1 diagnosis in a patient with typical CM1-like symptoms [3–5]. Recent recommendations suggest that a CM1 threshold of > 3 mm may suffice, especially if syringomyelia (SM) or peg-like cerebellar tonsillar deformation is present [6]. Some authors feel that CM1 and CM0 are continuous, rather than distinct, malformations [7, 8].

A diagnosis of Chiari malformation type 0 (CM0) has been recommended for patients with CM1-like symptoms, minimal or absent TH, and mediating factors like SM and small posterior cranial fossa (SPCF) traits [6, 9]. CM0 can be defined by a volumetrically small posterior cranial fossa (PCF), tonsils at or just below the FM, obliteration of the cisterna magna, and cervical SM [6]. However, if SM is absent, the other three criteria are nonspecific diagnostic criteria for CM0. Many people have tonsils positioned at or around the FM. Cisterna magna obliteration is common (8.4%). To improve CM0 diagnostic specificity in the absence of SM the study’s first aim is to establish reliable clinical and radiologic criteria for CM0 diagnosis [10]. Urbizu et al. proposed that two or three morphometric measures of PCF, not including TH, could help distinguish between CM1, borderline CM1, and CM1.5 with a 90% accuracy rate [5]. The borderline CM1 patient cohort included three patients with CM0-related SM [5]. Their method was only 54% to 71% accurate in diagnosing CM0 [5]. Their study suggested that the diagnosis of CM0 could potentially be made automatically using machine learning methods.

Our second aim is to create a predictive model for CM0. We hypothesize that the downward displacement of the brainstem and cerebellar tonsils is related to SM development in CM1 and CM0 patients [9, 11, 12]. Our study’s third aim is to identify MRI morphometric parameters of the PCF that are closely related to hindbrain descent, and to analyze their sensitivity and selectivity for predicting associated syringomyelia [7, 13].

Understanding the size and location of syringes is critical for discerning SM etiology and planning SM treatment [6, 14, 15]. Surgery may be recommended if the Vaquero index (the midsagittal greatest syrinx width divided by the associated spinal canal width) exceeds 0.5 in CM1 patients with SM [6]. The most common SM subtypes are CM1-related and idiopathic. Cases of idiopathic SM can be challenging to differentiate from CM0-related SM [16]. In one study, the mean midsagittal syrinx diameter was 2.7 ± 1.9 mm in 17 adult patients with CM0 and SPCF, which was significantly smaller than in primary CM1 patients (5.5 ± 4.7 mm) [17]. However, in 4 CM0 patients, syringes were wide like in CM1 [15]. In other studies, CM0 patients had various syrinx sizes [18, 19]. Our study’s **fourth** aim was to compare the syrinx sizes of CM0 and CM1 patients.

## Methods and materials

### Participants and clinical evaluation

The present study included adult symptomatic patients with primary CM1 and CM0-associated SM. All patients had MRI evidence of a longitudinally oriented, fluid-filled spinal cord cavity [6], a narrow cisterna magna [2, 10, 19], and SPCF characterized by a shortened clivus (SmCL pcf), supraocciput (SmSO pcf), or both (GlSm pcf) on the midsagittal pentagonal PCF outline [2, 20]. The clivus and supraocciput were considered short if their lengths were ≤ 40 mm [21, 22], one standard deviation below the control group’s mean. These control values were like those found in other studies [2, 23]. The cerebellar tonsil position relative to the FM was assessed on the midsagittal (T midsag), axial, and coronal MRI (T cor) images.

The patients were divided into two groups based on the maximum values of TH (T cor): *CM0-SM –* defined as SM and SPCF with the cerebellar tonsils extending from the inferior outlet of FM ≤ 2 mm (20 M/17F, 46 ± 15 years); *CM1-SM* – defined as SM and SPCF with one or both cerebellar tonsils extending ≥ 3 mm below the FM (24 M/22F, 49 ± 12 years). The control group (*Cntr*) was comprised of 25 subjects (14 M/11F, 43 ± 12 years) who underwent MRI for any reason and had normal brain and spinal cord imaging without central canal dilatation, SPCF features, or imperceptible cisterna magna. In controls, both cerebellar tonsils were positioned superior to the inferior outlet of the FM.

The study excluded patients with basilar invagination or other obvious osseous malformations of the cranio-vertebral junction, secondary TH (due to intracranial hypertension, spinal hypotension, or tethered cord syndrome), previous craniocervical decompression surgery, and age < 18 years.

Symptoms and signs of CM1 and SM-related central myelopathy were compared among the patient and сontrol groups. The Chiari Severity Index (CSI) assessed the clinical severity grade of the *CM0-SM* and *CM1-SM* patients [14].

### Radiological evaluation

**Fig. 1:**
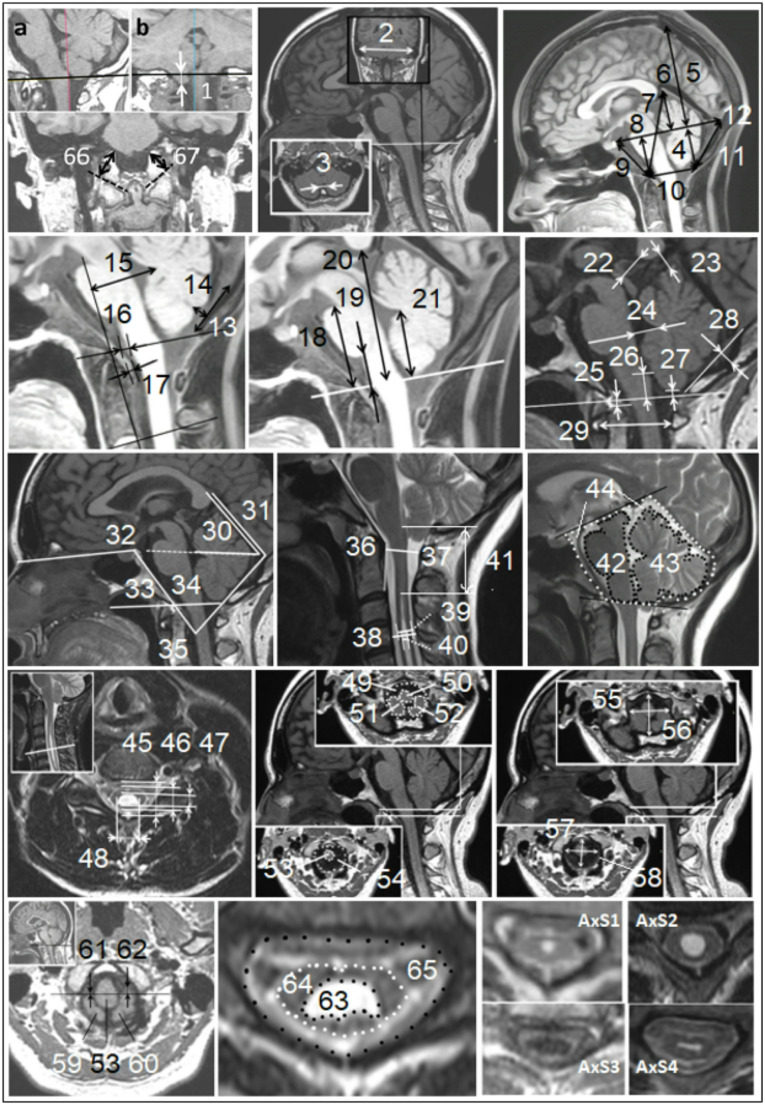
MRI-morphometric quantitative and qualitative variables. *The linear*,* angular*,* and area measurements*: 1-**T cor** – the grade of cerebellar TH below the level of the inferior outlet of FM on coronal T1 MRI (first, the FM level (black line) was determined on the sagittal image (**a**) in 3D MPR mode of the RadiAnt DICOM Viewer, and then **T cor** was measured from its projection on the coronal image (**b**)), 2**-X** - width of the PCF, 3-**B** - width of the cisterna magna, 4-**h** - posterior height of the osseous part PCF, 5-**H** - height of the skull above the Twining line, 6-**h eb** - height of the extrabone part PCF, 7-**Z** - height of the PCF, 8-**h ant** - anterior height of the osseous part PCF, 9-**CL** - clivus length, 10-**FM** - anteroposterior diameter of the foramen magnum on midsagittal MRI, 11-**SO** - supraocciput length, 12-**Y** - anteroposterior length of the PCF, 13-**A** - height of the cisterna magna, 14-**C** - depth of the cisterna magna, 15-**M-FVV** – distance between the M-line (Line M is drawn across the clivus vertex and perpendicular to the C-2 endplate) and fourth ventricle vertex, 16-**v** - anteroposterior width of the ventral subarachnoid space on the FM level, 17-**ROST** - anteroposterior distance of the soft tissue behind the odontoid process, 18-**P-FM**, 19-**M-FM**, 20-**C-FM**, 21-**F-FM** - distances from FM to pons, to medulla, to corpus callosum, and to fastigium, respectively, 22-**SC-ISCC**, 23-**VER-ISCC** - distances from the uppermost part of the superior colliculi and the uppermost part of the culmen of cerebellar vermis, respectively, to the inferior part of the splenium of corpus callosum, 24-**FV** - distance between the fastigium and the floor of the fourth ventricle, 25-**ChD** - distance between the Chamberlain line and tip of the dens axis (positive if the tip is above the line), 26-**obex-McR** - the distance between the obex and the basion-opisthion line, 27-**T midsag** - cerebellar tonsils herniation on the midsagittal MRI, 28-**h so** - depth of the supraocciput curvature, 29-**Apd C1** - distance between the inner surfaces of the anterior and posterior arches of the atlas, 30-**TTw** - angle at the intersection of the tentorium and the Twining line, 31-**TA** - angle formed by the tentorium and a line from the internal occipital protuberance to the opisthion, 32-**BA** - angle formed by the nasion, dorsum sella, and basion, 33-**CLgr.** – Clivus Gradient: angle of the clivus to the McRae’s line, continuing anteriorly from the basion, 34-**BoA** - Boogaard angle, 35-**C-SO** - angle between clivus and supraocciput, 36-**CAAm** - the modified clivoaxial angle: angle between the dorsal surface of the clivus and the posterior surface of the retro-odontoid soft tissue, 37-**apsc С1** and 38-**Sag apsc Smax** - anteroposterior diameters of the spinal canal at the level of C1 vertebrae and at the level of maximal syrinx on the sagittal MRI, respectively, 39-**Sag apscord Smax** and **40-Sag apd Smax** anteroposterior diameter of the spinal cord and of the syringomyelitic cavity at the level of maximal syrinx on the sagittal MRI, respectively, 41-**FVSD** - distance between fourth ventricle and uppers level of syrinx, 42-**BS-PCF**, 43-**C-PCF**, 44-**A-PCF** - the midsagittal brainstem and cerebellar areas in the PCF region, and the PCF region as a whole, respectively, **A-PCF** subdivided into extra-osseous part and osseous part, located, respectively, above and below the Twining line (**S pcf eb** and **S pcf b**, not shown); 45-**Ax apsc**, 46-**Ax apscord**, 47-**Ax apd Smax**, 48-**Ax trd Smax** - anteroposterior diameters of the spinal canal, spinal cord, syringomyelitic cavity, and transverse diameter of the syringomyelitic cavity, respectively, at the level of maximal syrinx on the axial MRI, 49-**S fm sot**, 50-**S m sot**, 51-**S t dex sot**, 52-**S t sin sot** - areas of the FM, the medulla, the right tonsil, and the left tonsil, respectively, at the level of the superior outlet of FM, 53-**S m iot**, 54-**S fm iot -** areas of the medulla and the FM, respectively, at the level of the inferior outlet of FM, 55-**apd FM sot**, 56-**trd FM sot** - anteroposterior and transverse diameters of the FM at the level of the superior outlet of FM, respectively, 57-**apd FM iot**, 58-**trd FM iot** - anteroposterior and transverse diameters of the FM at the level of the inferior outlet of FM, respectively, 59-**S t dex iot**, 60-**S t sin iot** - areas of the right and the left tonsils, respectively, at the level of the inferior outlet of FM, 61-**VHd** and 62-**VHs** – ventral herniation of the right and left cerebellar tonsils, respectively, were determined on axial images at the FM level by the length of the displacement of the right and left tonsils in the ventral direction from the level of the transverse diameter of the medulla oblongata, 63-**S syr max**, 64-**S sc Smax**, 65-**S vc Smax** - syrinx area, spinal cord area, and vertebral canal area, respectively, at the level of maximal syrinx on the axial MRI, 66-**Exoc dex**, 67-**Exoc sin** – the axial length of the right and left exo-occiput, respectively (from the top of the jugular tubercle to the bottom of the occipital condyle). *Qualitative variables* (axial syrinx phenotypes): **AxS1** - rounded with diameter no more than 3 mm, **AXS2** - rounded with diameter of more than 3 mm, **AxS3** – “large” oval with an anteroposterior diameter of more than 3 mm, **AxS4** - “flattened” or collapsed oval with a transverse diameter of more than 3 mm, and anteroposterior - less than 3 mm (flattened on axial and filiform on midsagittal images)

Patients were imaged using 1.5 T MRI scanners (EXCITE, GE Healthcare, Waukesha, Wisconsin). A trained researcher (A.F.) analyzed the images, with measurements presented in Fig. 1.

#### Calculated variables

Evans’ index (EI) > 0.3 signified ventricular dilatation [24]. The ratio of the height of the intracranial spaces below the Twining line (h, the osseous part of the PCF) and above this line (H) was calculated as h/H [11]. The volume of the PCF in cm3 (V pcf) was calculated using the formula: [4/3 × π×(X/2 × Y/2 × Z/2)], where X, Y, Z are, respectively, the width, anteroposterior length, and height of the PCF. The volume of the cisterna magna (V cm) was calculated using the formula [10]: [A× B ×C]/6, where A, B, and C are, respectively, the height, width, and depth of the cisterna magna. The PCF crowdedness index (PCF CI) was calculated using the formula: [(BS-PCF + C-PCF)/A-PCF] [25]. The midsagittal area of the PCF’s cerebrospinal fluid (CSF) spaces was calculated as CSF-PCF = [A-PCF - BS-PCF - C-PCF] [25]. Sagittal taper PCF was calculated as (Y- FM)/((h + h ant)/2). Coronal taper PCF was calculated as (Х- trd FM iot)/((h + h ant)/2). Up PCF was derived from the sum of VER-ISCC + SC-ISCC and was called 1 if < 11, 2 if 11–17, and 3 if > 17. Indicators evaluated at the level of the inferior outlet of FM included [26]: area of the tonsils (S tt iot = S t dex iot + S t sin iot); the quotient of tonsil area to the total FM area (S tt iot/S fm iot); CSF area (S csf iot = S fm iot - S m iot - S tt iot); the proportion of the FM inferior outlet occupied by CSF spaces (the inverse of the FM-crowdedness index) - S csf/S fm iot; and the FM-crowdedness index or the neural element occupancy of the inferior outlet of the FM ((S m + S tt)/S fm iot). Indicators at the level of the superior outlet of FM [26] were calculated similarly. The syrinx was measured at its widest point. Absolute linear and area measurements (shown in Fig. 1) and relative indicators were also evaluated, including two on the axial MRI (S syr max/S vc max, Ax apscord/Ax apsc) and two on the sagittal MRI (Sag apd Smax/Sag apscord Smax, Vaquero Index = Sag apd Smax/Sag apsc). Syrinx length was measured by the number of vertebral segments the syrinx spanned. The level of the widest part of the syringomyelia cavity was described by the number of vertebral segments from the FM (1, if C1; 7, if C7; 8, if Th1). The rostral level of the SM cavity (1, if it is above C1; 2, if at C1; 3, if at C2; 9, if at Th1) was one number higher than the vertebral segments from the FM.

#### Qualitative variables

Four syringomyelia cavity phenotypes were identified on axial images at the widest part of an SM cavity (AxS1 – AxS4; Fig. 1). Sagittal MRI revealed three syrinx configurations: (1) distended - long and wide, (2) slender - long and narrow, and (3) circumscribed - limited to 2–3 contiguous vertebral segments. Ventral tonsillar herniation (VH) was defined as one or both tonsils crossing anterior to a transverse line bisecting the caudal medulla at the FM [27]. The VH index was 0 if the cerebellar tonsils were dorsal to this line, 1 if one tonsil partially extended ventral to this line, and 2 if part of both tonsils lay ventral to this line (“suffocating hugs”).

The MRI parameters were organized into eight categories directly or indirectly assessing PCF anatomic structures and spinal syringes: I, Size of the PCF; II, Shape of the PCF; III, FM size and cranio-vertebral junction; IV, Tonsillar and hindbrain descent; V, PCF CSF spaces and PCF crowdedness; VI, Crowdedness within the FM; VII, Syrinx size, and VIII, Other. The Group I and II parameters assessed SPCF; the Group III - caudal hindbrain displacement and FM crowdedness; the Group IV-VII - discrepancy between the dimensions of the SPCF and its contents, and the consequences of this discrepancy [7].

### Statistical analysis

Categorical variables are shown as percentages, and continuous variables as means with standard deviations. Comparisons between patients and controls were made using the U-test and chi-square test, with *p* < 0.05 considered significant (IBM SPSS Statistics 23, Armonk, NY). Spearman’s nonparametric rank correlation assessed relationships between variables. The analysis used PCF morphometric parameters other than TH, CL, SO, and narrow cisterna magna to develop a predictive model for symptomatic CM0 and CM1. Binary logistic regression identified key variables differentiating patients and controls (Table 1), with model accuracy determined via ROC analysis.

**Table 1:** The initial parameter lists to create predictive models

	I. Size of the PCF	18	Apd С1
1	h ant	19	apd FM iot
2	h		IV. Tonsillar and hindbrain descent
3	S pcf	20	C-FM
4	Z	21	M-FM
5	Y	22	P-FM
6	X	23	F-FM
	II. Shape of the PCF	24	obex-McR
7	C-SO		V. PCF liquor spaces and PCF crowdedness
8	BoA	25	BS-PCF
9	TA	26	A-PCF
	III. FM size and cranio-vertebral junction	27	VER-ISCC
10	apd FM sot	28	SC-ISCC
11	S fm sot	29	M-FVV
12	ROST	30	v
13	CAAm	31	C-PCF
14	ChD		VI. Crowdedness within the FM
15	apsc С1	32	S csf iot
16	S fm iot	33	S csf sot
17	FM	34	S m iot

## Results

### Clinical findings

There was no significant difference in the age of onset, duration of symptomatic disease, or mean Chiari Severity Index (CSI) between the CM0-SM and CM1-SM groups (Table 2). However, grade 2 CSI was more common in CM0 patients (*p* < 0.005).

**Table 2:** Clinical and qualitative morphometric characteristics in *Primary CM0-SM*,* primary CM1-SM*, and control groups

Parameters	CM0-SM	CM1-SM	Cntr
Clinical characteristics			
Age of clinical manifestation, y	29 ± 18	33 ± 15	33 ± 4
Duration of symptoms, y	18 ± 14	16 ± 14	8 ± 7
Paresis, %	70*	65*	0
Segmental-dissociated sensory loss, %	70*	86*	0
Pyramidal signs, %	84*	91*	8
All sensory disturbances, %	94*	98*	4
No headache, %	37	37	43
non CM1-associated headache, %	40	26	39
CM1-associated headache (%), including:	23	37	17
- cough headache, %	10	14	0
CSI, mean	2.11 ± 0.70	2.09 ± 0.89	
CSI 1, %	19	35	
CSI 2, %	51**	22	
CSI 3, %	30	43	
Qualitative morphometric characteristics			
SmSO pcf	32% *	39% *	0%
SmCL pcf	38% * **	15% *	0%
GlSm pcf	30% *	46% *	0%
normal pcf	0% *	0% *	100%
Peg-like deformation of the tonsillar profile	11% **	78% *	0%
Syrinx configuration on sagittal MRI:	13% **	35%	
- Distended type			
- Slender type	65%	56%	
- Circumscribed type	22%	9%	
Syrinx configuration on axial MRI:	11%	2%	
- AxS1			
- AxS2	11%	17%	
- AxS3	11% **	33%	
- AxS4	67% **	48%	
FVSD < 11 mm	11%	11%	

* *p* < 0.05 vs. *Cntr*; ** *p* < 0.05 vs. *CM1-SM*

### MRI data

The mean values and characteristics for the eight groups of MRI indicators are shown in Tables 2, 3 and 4. In all groups, the cerebellar tonsils’ inferior extent was lower on the coronal (T cor) than the mid-sagittal MRI image (T midsag). Syringes in CM0 patients were significantly smaller than in CM1 patients. 78% of patients with CM0 and 81% with CM1 had oval syringes with wider transverse than anteroposterior diameters.

**Table 3:** Quantitative parameters of the PCF in *Primary CM0-SM, Primary CM1-SM* and control groups: linear (in mm), angular (in degrees), area (in mm^2^), volume and indices

Variables	CM0-SM		CM1-SM		Cntr		*p* < 0.05
	M	m	M	m	M	m	
I. Size of the PCF							
SO	38.62	3.59	37	3.71	43.4	3.77	§ * **
S pcf b	2703.59	361.16	2593.72	311.01	3286.23	405.4	* **
h ant	30	3.93	28.63	3.25	35.52	3.07	* **
h	28.49	4.34	27.11	4.36	34.52	4.49	* **
h/H	0.34	0.06	0.33	0.07	0.44	0.08	* **
S pcf	3857.24	388.55	3830.28	353.47	4444.23	434.9	* **
CL	38.92	4.25	38.61	3.24	43.16	2.75	* **
Z	61,85	5,61	61,3	4,88	66,24	3,78	* **
V pcf, cm^3^	276.31	38.27	279.89	34.25	298.89	29.77	* **
H	82.6	7.57	82.58	7.81	79.4	6.87	*
h so	2.32	2.72	2.15	2.72	2.88	1.86	
Y	82.46	6.48	84.17	4.9	83.4	4.05	
S pcf eb	1153.65	226.37	1236.55	206.66	1158	181.24	§
h eb	28	4.96	29.35	4.43	27.76	4	
X	103.05	5.06	103.17	5.02	102.92	4.47	
Exoc dex	1.92	0.30	1.79	0.26	2.26	0.25	* **
Exoc sin	1.93	0.29	1.79	0.23	2.19	0.30	* **
II. Shape of the PCF							
Coronal taper PCF	2.54	0.35	2.61	0.46	2.05	0.25	* **
C-SO	79.51	13.29	82	9.58	68.48	7.22	* **
Sagittal taper PCF	1.85	0.39	1.97	0.43	1.52	0.18	* **
BoA	128.59	7.92	131.11	6.74	124.08	5.99	* **
TA	87.78	**10.17**	85.35	9.89	89.84	6.45	**
III. FM size and cranio-vertebral junction							
apd FM sot	36.22	3.04	38.72	3.69	40	2.52	§ *
S fm sot	985.39	165.46	1071.39	196.51	1146.77	173.83	§ *
ROST	4.14	1.18	4.04	1.15	3.12	1.05	* **
CAAm	140.57	9.67	140.07	9.29	146.4	8.7	* **
trd FM sot	34.36	3.55	34.96	4.19	36.36	3.74	
ChD	0.51	2.29	1.22	1.62	−0.72	2.54	**
apsc С1	16.32	2.11	17.13	2.33	17.16	2.75	
trd FM iot	30.58	3.87	31.67	3.66	31.64	3.03	
S fm iot	720.52	151.98	761.03	150.95	765.18	126.7	
FM	34.97	2.43	35.48	2.55	35.6	2.92	
Apd С1	31.76	2.07	32.22	2.12	32.12	2.07	
apd FM iot	30.3	3.91	30.5	3.28	30.64	2.66	
IV. Tonsillar and hindbrain descent							
T midsag	−0.94	2.59	6.5	3.58	−3.76	2.91	§ * **
T cor	0.7	0.74	7.74	2.71	0.2	0.41	§ * **
C-FM	54.52	3.55	54.18	3.26	58.8	3.62	* **
M-FM	11.54	2.83	9.54	3.12	13.52	2.45	§ * **
P-FM	36.11	3.63	34.93	2.87	39.04	3.42	§ * **
F-FM	27.67	3.04	26.41	2.91	29.56	2.57	* **
obex-McR	5.86	2.95	−0.52	5.15	7.64	1.7	§ * **
V. PCF liquor spaces and PCF crowdedness							
V cm, mm^3^	113.36	189.16	22.6	50.66	299.37	204.78	§ * **
BS-PCF	8.85	0.79	9.15	0.74	10.16	0.8	* **
A-PCF	31.77	2.49	32.81	2.52	35.39	2.82	* **
VER-ISCC	6.84	1.95	6.59	1.96	10	2.43	* **
CSF-PCF	9.53	1.94	9.54	2.04	12.05	2.03	* **
PCF CI	0.7	0.05	0.71	0.05	0.66	0.04	* **
up PCF	1.94	0.57	2.02	0.4	2.24	0.44	* **
SC-ISCC	6.45	2.47	6.68	2.08	5.64	1.44	**
M-FVV	29.08	4.75	27.93	4.32	30.16	4.37	
v	6.25	2.21	4.96	1.88	6.68	1.97	§ **
C-PCF	13.4	1.98	14.11	1.73	13.17	1.4	**
FV	9.44	2.43	9.83	2.64	9.36	1.35	
VI. Crowdedness within the FM							
VH index	0.72	0.63	0.61	0.81	0.12	0.44	* **
VHs	1.59	2.01	1.39	2.08	0.08	0.4	* **
S tt/S fm iot	9.32	12.59	35.54	11.78	1.59	3.63	§ * **
S tt iot	71.42	95.98	270.67	114.14	13.45	30.99	§ * **
S csf iot	528.14	144.52	343.42	128.37	627.77	115.69	§ * **
S csf/S fm iot	73.96	12.87	45.3	13.29	81.97	5.69	§ * **
S tt sot	348.49	117.88	521.15	150.84	409.43	139.4	§ **
S csf sot	478.42	158.15	382.58	143.42	565.16	153.36	§ **
S m sot	156.88	29.11	169.52	38.86	172.19	37.85	
VHd	0.31	0.82	0.66	1.46	0.16	0.55	
S m iot	116.85	27.14	144.24	41.71	123.96	27.13	§ **
S tt/S fm sot	35.48	11.3	48.48	11.16	35.4	10.11	§ **
S csf/S fm sot	48.24	12.24	35.5	10.88	49.32	11.62	§ **
VII. Syrinx size							
Ax trd Smax	4.93	2.88	7.88	4.41			§
FVSD	61.22	55.03	29.28	19.12			§
S syr max	0.14	0.22	0.37	0.46			§
Sag apd Smax	2.57	1.99	4.57	3.34			§
S syr max **/** S vc Smax	5.55	7.49	12.39	13.17			§
Vaquero Index	0.18	0.14	0.31	0.22			§
The rostral level of the syrinx	4.73	3.03	3.11	1.32			§
Sag apscord Smax	7.35	1.9	9	2.9			§
Sag apd Smax/Sag apscord Smax	0.33	0.18	0.46	0.23			§
Ax apscord	7.76	2.43	9.22	2.92			§
Ax apd Smax	2.89	2.32	4.39	3.17			§
Length of Syrinx	10.14	6.4	12.22	5.31			
Sag apsc Smax	14.16	1.89	14.54	2.2			
VIII. Others							
EI	0.27	0.03	0.28	0.03	0.25	0.02	* **
CLgr.	51.41	7.92	48.89	6.74	55.92	5.99	* **
ВА	118.91	8.08	117.39	5.65	114.68	5.16	*
HW	36.28	3.8	38.23	5.01	34.16	3.17	* **
S vc Smax	2.33	0.56	2.75	0.87			§
Ax apsc	14.95	3.05	14.74	2.13			

§ *Primary CM0-SM vs. Primary CM1-SM*; *Primary CM0-SM vs. Cntr; **Primary CM1-SM ver. Cntr

**Table 4:** Comparative characteristics of MRI morphometric parameters in *Primary CM0-SM*, *Primary CM1-SM*, and control groups

Parameters	CM0-SM vs Cntr	CM1-SM vs Cntr	CM1-SM vs CM0-SM
I. Size of the PCF			
h, h/H, h ant., Z, CL, SO, S pcf, S pcf b	↓↓*	↓↓	↓ (↓↓ for SO)
V pcf	↓↓	↓↓	↑
S pcf eb	↓	↑	↑↑
II. Shape of the PCF			
BoA, C-SO	↑↑	↑↑	↑
TA	↓	↓↓	↓
SmCL pf, %			↓↓
III. FM size and cranio-vertebral junction			
apd FM sot, S fm sot	↓↓	↓	↑↑
ChD	↑	↑↑	↑
ROST	↑↑	↑↑	↓
CAAm	↓↓	↓↓	↓
IV. Tonsillar and hindbrain descent			
T cor, T midsag	↑↑	↑↑	↑↑
VH index, VHs	↑↑	↑↑	↓
obex-McR, M-FM, P-FM, C-FM, F-FM	↓↓	↓↓	↓↓ (↓ for C-FM, F-FM)
V. PCF liquor spaces and PCF crowdedness			
VER-ISCC, CSF-PCF, V cm, v	↓↓ (↓ for v)	↓↓	↓↓ (↓ for VER-ISCC, CSF-PCF)
up PCF, A-PCF, BS-PCF	↓↓	↓↓	↑
SC-ISCC, C-PCF, PCF CI	↑ (↑↑ for PCF CI)	↑↑	↑
VI. Crowdedness within the FM			
S csf sot, S csf/S fm sot	↓	↓↓	↓↓
S tt sot, S tt/S fm sot	↑ (↓ for S tt sot)	↑↑	↑↑
S csf iot, S csf/S fm iot	↓↓	↓↓	↓↓
(S m + S tt)/S fm iot	↑↑	↑↑	↑↑
S tt iot, S tt/S fm iot, S m iot	↑↑ (↓ for S m iot)	↑↑	↑↑
VII. Syrinx size			
Sag apd Smax, Ax apd Smax, Ax trd Smax, Vaquero Index, S syr max,Distended type, S syr max/S vc max,Sag apd Smax/Sag apscord Smax, Sag apscord Smax, Ax apscord			↑↑
AxS3, FVSD, rostral level of the SM cavity			↓↓
VIII. Others			
EI, HW	↑↑	↑↑	↑
CLgr.	↓↓	↓↓	↓
ВА	↑↑	↑	↓

* ↓↓ or ↑↑ - p < 0.05; ↓ or ↑ - statistically insignificant trends

Correlations between clinical and MRI factors are shown in Table 5 and Suppl. Table I. Correlations among MRI indicators appear in Suppl. Tables II-XII. In CM1 patients, TH indices (T midsag and T cor) correlated with the age of onset of clinical manifestations and proportion of CSI grade 2, respectively. In CM0 and CM1 patients, the TH indices were not associated with CM1 and SM symptoms. In the combined group of CM0-SM and CM1-SM patients, the syrinx cavity size (S syr max) inversely correlated with the CSF proportion of the FM inferior outlet area (S csf/S fm iot;*r*= −0.326,*p*= 0.004) and obex position (obex-McR;*r*= −0.321, *p*= 0.003), and directly correlated with T cor (*r*= 0.394, *p*= 0.001).

**Table 5:** Clinical-MRI correlation (*p* < 0.05) in *Primary CM0-SM* and *Primary CM1-SM* groups

Parameters	Age of clinical manifestation	Paresis	Segmental-dissociated sensory loss	CM1-associated headaches	CSI
I. Size of the PCF	CM0-SM	CM0-SM CM1-SM	-	-	CM0-SM CM1-SM
II. Shape of the PCF	CM0-SM	CM0-SM CM1-SM	-	CM0-SM	
III. FM size and cranio-vertebral junction	CM0-SM CM1-SM	CM0-SM	CM0-SM CM1-SM	CM1-SM	
IV. Tonsillar and hindbrain descent	CM1-SM	-	-	-	CM1-SM
V. PCF liquor spaces and PCF crowdedness	-	-	CM1-SM	CM0-SM	
VI. Crowdedness within the FM	CM0-SM	-	CM0-SM	CM0-SM CM1-SM	CM0-SM
VII. Syrinx size	-	CM0-SM CM1-SM	CM0-SM CM1-SM	CM0-SM CM1-SM	CM0-SM CM1-SM

## Development of a probability mode

The predictive model to differentiate the PCF of CM0 patients from the control group included three variables: VER-ISCC, h ant, and apd FM sot, in the equation: P(case) = 1/(1 + exp(-y)), where y = 35.47–0.55 * VER-ISCC − 0.4 * h ant − 0.47 * apd FM sot. Using a cut-off of *P* = 0.4, this model had 90% sensitivity and 84% specificity, with an AUC (Area Under the Curve) of 0.954. For CM1-SM patients, the sensitivity was 86%. The model for PCF CM1-SM vs. PCF control included the variables obex-McR and h ant in the equation: P(case) = 1/(1 + exp(-y)), where y = 28.763 − 0.603 * obex-McR − 0.82 * h ant. With a cut-off of *P* = 0.58, this model had 94% sensitivity, 96% specificity, and an AUC of 0.986. The sensitivity for CM0-SM patients vs. controls was 59%.

## Discussion

### The morphometric characteristics of the PCF in CM0 and CM1 patients

The morphometric characteristics of the PCF in CM0 and CM1 patients are compared to those of controls in Tables 2, 3 and 4. In both the CM0-SM and CM1-SM groups, alterations from controls included a reduced, flattened, and overcrowded PCF, more pronounced in CM1 than in CM0. The area (S fm sot) and anteroposterior diameter (apd FM sot) of the FM were significantly smaller in CM0 patients than in CM1 patients and controls, which did not differ from each other. This reduction may explain the lack of cerebellar tonsil protrusion in CM0 patients (Table 3).

### The relationship of cerebellar tonsillar position to FM crowding in CM0 and CM1 patients

In CM0 and CM1 patients, the TH grade directly correlated with FM occupancy parameters (S tt iot, S tt sot) and inversely correlated with CSF space at the FM (Supplemental Table II). The indices of superior FM outlet crowdedness, such as S csf sot and S csf/S fm sot, were lower in CM1 patients than in CM0 patients and controls, and there were no significant differences in these indices between CM0 patients and controls (Table 4). CM0 patients had significantly smaller absolute values for both the diameter and cross-sectional area of the FM superior outlet compared to the CM1 and control groups (Table 4). Our findings align with reports of small inferior and superior outlets in CM1 patients with TH < 5 mm. Milhorat and coauthors described reduced inferior and superior FM outlet areas in CM1 patients compared to controls [26]. In CM0, other factors like diminished PCF height also contributed to FM overcrowding, explaining how less than 5 mm of TH can yield symptoms like those of CM1. CM0 and CM1 have distinct reasons for FM overcrowding: a small FM area in CM0 and tonsillar herniation in CM1.

### The relationship between obex position and FM crowding in CM0 and CM1 patients

This study analyzed the distance between the obex and the FM. In CM1 patients, this distance was − 0.52 ± 5.15 mm (below the FM), significantly lower than in the CM0 and control groups. In the CM0 syringomyelia (CM0-SM) group, the distance was 5.86 ± 2.95 mm (above the FM), which was significantly closer to the FM than in controls (see Table 4). In CM1 patients, obex descent correlated with several anatomical features, while in CM0 patients, it correlated with the cerebellar tonsil area and other measurements (Suppl. Table II). CM0 patients also showed that obex descent correlated with the Evans index-based hydrocephalus grade (Suppl. Table II). However, in both CM1-SM and CM0-SM groups, the Evans index did not correlate with TH (Suppl. Table III). In both CM0 and CM1 patients, the height of the superior cerebellar cisterns (up PCF) was significantly lower than in controls, and narrower cisterns in CM1 patients were associated with more caudal obex positions [28] (Suppl. Table II). In CM0 patients, low obex position correlated with features like ventriculomegaly and hindbrain descent, while in CM1 patients, low obex position correlated with TH and measures of caudal displacement (Suppl. Table II).The occipital bone is formed from paraxial mesoderm of the first four somites, while the rostral skull develops from cephalic mesoderm. The supratentorial part of the occipital bone develops from intramembranous bone growth that does not require an intermediary cartilage stage [29]. The infratentorial part of the occipital bone develops by replacing cartilage within the sclerotomes of the first four embryonal somites with bone. The first two occipital sclerotomes form the basiocciput, which includes the occipital condyles and the inferior part of the clivus located anterior and superior to the foramen magnum. The basiocciput joins the paired exoccipital bones at the bilateral basiexoccipital synchondroses, the sphenoid bone at the sphenooccipital synchondrosis, and the petrous temporal bones at the bilateral petrooccipital fissures. The third occipital sclerotome forms the exoccipital bones located posterior to the occipital condyles and on the lateral sides of the foramen magnum. All four occipital sclerotomes contribute to the occipital bone (supraocciput) and posterior parts of the foramen magnum. The process of posterior fossa development is complex and regulated by transcription factors that sequentially activate and suppress genes within the occipital sclerotomes. Genetic and epigenetic factors influence the development of the basioccipital bone [30]. We speculate that genetic and epigenetic factors contributed to the reduced FM diameter seen in CM0 patients, reduced supraocciput length seen in CM1 patients, and reduced PCF volumes shared by the CM0 and CM1 patients. Other anatomical measures associated with a caudal hindbrain position.

In CM0 and CM1 patients, various measurements from hindbrain landmarks to the FM correlated with characteristics of a smaller and flattened PCF (Suppl. Tables VI-VII). Previous research indicated that CM1 patients exhibited significantly smaller FM outlet diameters than controls [26]. In this study, PCF volume and size did not correlate with FM osseous dimensions but did correlate with parameters like BS-PCF and the cross-sectional area of the medulla at the FM level (Suppl. Table VIII). Notably, reduced FM size and surrounding PCF volume were associated with primary CM0 pathogenesis [31].

The C-SO angle was wider in CM0 and CM1 patients than in controls (Table 4) and correlated with FM size and CSF space reduction. The tentorial angle (TA) was wider in CM1 than CM0 patients, and inversely correlated with FM CSF space in both groups. Overall, FM crowdedness was more closely related to FM size and PCF geometry than PCF volume [31]. In our analysis of primary CM0 and CM1, we found that a smaller volume and size of the PCF correlated with a lower position of the pons, corpus callosum, and medulla, as measured by P-FM, C-FM, and M-FM. However, reduction in PCF volume and size did not correlate with inferior positioning of the cerebellar tonsils and obex, which are assumed to contribute to crowding of the cranial outlet. The height of the anterior part of the PCF (h ant) was closely linked to hindbrain descent. In CM0, h ant correlated with the distance between the obex and foramen magnum. In both CM0 and CM1, h ant correlated with P-FM, C-FM, and M-FM measurements [5, 31].

For CM1, h ant was associated with FM crowding parameters. Overall, h ant was a strong predictor of hindbrain descent and could aid in diagnosing CM1 spectrum disorders, especially when tonsillar herniation is less than 5 mm. These results imply that reduced PCF volume and size lead to caudal displacement of hindbrain structures that may not extend below the FM. The flattening of the PCF and FM size impacted this caudal displacement. Using a logistic regression model, we identified key MRI measures for diagnosing CM0 and CM1. The combination of three PCF measurements—’ h ant,’ ‘VER-ISCC,’ and ‘apd FM sot’—achieved a sensitivity of 90% and specificity of 84% for identifying CM0. Combination of two PCF measurements — ’ h ant,’ and ‘obex-McR’ — yielded a sensitivity of 94% and specificity of 96% for identifying CM1 patients with SM, regardless of TH amount.

### The morphometric PCF features associated with Syrinx formation in CM0 and CM1 patients

Earlier studies were inconsistent in their findings as to which PCF morphometric measures best predicted the presence or absence of SM in CM1 spectrum disorder patients [32]. The relationship between the extent of TH and SM is unpredictable. The capacity of the syrinx filling mechanisms may influence the cross-sectional area and volume of the syrinx [12, 15]. Larger syrinx size increases the risk of myelopathy and neurological progression [14, 33].

In the present study, the SM cavity in CM0 patients was significantly less expanded and found more caudally than in CM1 patients (Table 4). All measurements characterizing syrinx size were statistically smaller in CM0 than CM1 patients (Table 4). Our results correspond with the opinion of CM1 experts who consider narrower syringes to be typical of CM0 [3].

In our *CM0-SM* and *CM1-SM* groups, TH amount (T cor, T midsag) and FM crowdedness (S tt iot, S tt sot, S csf/S fm iot, S csf/S fm sot) did not correlate with any syrinx size measure (Sag apd Smax, Ax apd Smax, Ax trd Smax, S syr max, Sag apd Smax/Sag apscord Smax, Vaquero Index) (Suppl. Table XII). In a group made up of all study patients with primary CM0-SM and primary CM1-SM, syrinx cavity size (S syr max) inversely correlated with the proportion of the FM inferior outlet occupied by CSF spaces (S csf/S fm iot; *r*=−0.326, *p* = 0.004) and obex position (obex-McR)(*r*=−0.321, *p* = 0.003) and correlated with TH (T cor; *r* = 0.394, *p* = 0.001). The correlative analysis demonstrated larger syrinx size in CM1 (Ax apd Smax, S syr max/S vc max, Vaquero Index, Sag apd Smax, Sag apd Smax/Sag apscord Smax,) than in CM0. Syrinx length was associated with a lower obex position (Suppl. Table II).

One study described SPCF morphometric features that predicted which cases of cervical SM were related to CM0 [17]. CM0-related SM is typically found centrally in the cervical spinal cord and has an oval or flattened syrinx shape (AxS3, AxS4; Fig. 1). In contrast, the incidental finding of a dilated spinal cord central canal is also centrally located but is round and usually in the thoracic spinal cord [34].

In a study by Hatano and colleagues, the FVSD (fourth ventricle to syrinx distance) was 7.5 mm in patients with FM arachnoiditis and SM versus 29.9 mm in patients with CM1 and SM [35]. In our present study, 11% of patients in the CM0 and CM1 groups had FVSD < 11 mm. The mean FVSD was 61.22 ± 55.03 mm in the *CM0-SM* group and 29.28 ± 19.12 mm in the *CM1-SM* group (*p* < 0.001). Occipital bone hypoplasia can also increase the chances of CM0-associate SM.

In our present study, syrinx size was smaller in CM0 than in CM1 patients. CM1 patients, compared to CM0 patients had a trend toward a smaller area of the PCF below the Twining line (S pcf b) [31] and smaller FM CSF areas (S csf iot; S csf sot; S csf/S fm iot; S csf/S fm sot) (Tables 3 and 4). The CSF area was narrowed by the herniated cerebellar tonsils (S tt iot; S tt sot; S tt iot/S fm iot; S tt sot/S fm sot) and medulla (S m iot; S m sot) (Tables 3 and 4) within the FM. FM obstruction is critical to CM0 and CM1 syrinx formation. Syringes may be smaller in CM0 patients than in CM1 patients because CM0 patients have less severe FM obstruction than CM1 patients [12].

### The key MRI measures for diagnosing primary CM0 and CM1

In our study, we found that the basiocciput (CL) and exo-occiput (Exoc dex, Exoc sin) were significantly shorter in both the CM0 and CM1 groups compared to the control group, with no difference between the CM0 and CM1 groups (Table 3). Additionally, the supraocciput (SO) was also shorter in CM0 and CM1 than controls. SO was significantly shorter in CM1 compared to CM0 (Table 3).

Anterior PCF height (h ant), measured from the basion to the Twining line, was reduced in both the CM0 and CM1 subtypes. Correlation analysis showed that ‘h ant’ positively correlated with PCF size, Twining angle, height, and hindbrain position markers, and negatively correlated with markers of PCF flattening (Suppl. Table II). Thus, ‘h ant’ may reflect basion position variations in CM1 spectrum disorders [5]. A genetic analysis of primary CM1 patients with short ‘h ant’ distances indicated downregulation of specific gene expression pathways [36]. Our findings linked CM0 to PCF crowding and reduced FM diameter, both of which hinder cerebellar tonsil prolapse. A combination of ‘obex-McR’ with ‘h ant’ accurately predicted primary CM1, while ‘h ant,’ ‘VER-ISCC,’ and ‘apd FM sot’ predicted primary CM0.

### Limitations

This present study utilized MRI, which is less ideal than CT for measuring bony structures. On CT, the greater attenuation of the X-ray beam by bone than by brain or CSF delineates bone architecture. On MRI, cortical bone appears as low-intensity and is more challenging to delineate from surrounding structures than on CT, which may introduce measurement errors. The length of the basiocciput, exo-occiput, and supraocciput can be measured more confidently by CT than by MRI because of this. Furthermore, magnetic field inhomogeneities of 1–3 mm outside the MRI scanner’s magnetic field isocenter may distort MRI measurements.

Several key findings about the pathogenesis and classification of CM1 and its subtypes have emerged from studies combining CT and MRI data [20, 25, 26, 31, 37]. The present study was not designed to include CT imaging of the skull base [26, 31, 37]. Many authors have reported that the primary and consistent patterns of PCF hypoplasia and overcrowding seen in CM0 and CM1 are detectable on MRI [2, 4, 5, 7, 20, 21, 23, 27, 32]. The features of our study data, obtained solely from MRI measurements, were consistent with published findings from studies using combined CT and MRI measurements, and supported the differentiation of CM0 from CM1 [31, 37].

## Conclusion

Primary CM1 spectrum disorders include CM1 and CM0, which share features of PCF hypoplasia, size reduction, and flattening. CM1 has more pronounced PCF hypoplasia, narrower FM pathways, and larger syringes than CM0. The inferior displacement of hindbrain structures contributes to neural crowding in both CM1 and CM0. The downward movement of hindbrain structures correlated with PCF flattening but not PCF size. CM0 was predicted by combining reduced h ant, PCF crowdedness (reduced VER-ISCC), and FM crowdedness (reduced apd FM sot). CM1 was predicted by combining reduced h ant and a caudal obex position (obex-McR). Our study identified four PCF morphometric markers (reduced h ant, reduced VER-ISCC, reduced apd FM sot, and caudal obex position (obex-McR) that predicted CM0 and CM1, warranting further prospective validation.

## Supplementary Information

Below is the link to the electronic supplementary material.

Supplementary Material 1

## Data Availability

All data from this study are in the published article and supplementary files.

## Abbreviations

AUC: Area under the curve
CM0: Chiari malformation type 0. (Diagnostic criteria of CM0 included typical Chiari I malformation symptoms and the cerebellar tonsils extending ≤ 2 mm inferior to the inferior FM outlet.)
CM0-SM: CM0 and syringomyelia. (The Primary CM0-SM group included patients with SM, SPCF, and cerebellar tonsils extending ≤ 2 mm inferior to the FM outlet.)
CM1: Chiari I malformation
CM1-SM: CMI and syringomyelia. (The Primary CM1-SM group included patients with SM, SPCF, and one or both cerebellar tonsils extending ≥ 3 mm below the FM.)
Cntr: Control group
CSF: Cerebrospinal fluid
CSI: Chiari Severity Index
FM: Foramen magnum
PCF: Posterior cranial fossa
SM: Syringomyelia
SPCF: Small posterior cranial fossa
TH: Tonsillar herniation
VH: Ventral tonsillar herniation
