# Supplementary material for: MRI-morphometric characterization of Chiari malformation types 0 and 1 with syringomyelia: implications for diagnosis and pathogenesis
Source: Neurol Sci. 2026 Mar 13;47(4):343. doi: 10.1007/s10072-026-08820-z (PMC12987787; doi:10.1007/s10072-026-08820-z)
Supplement: Supplementary file 1 — Supplementary Material 1 [file 10072_2026_8820_MOESM1_ESM.docx]

*Supplementary Information*

Suppl. Table I. Positive and negative correlations (*p*<0.05) between main clinical characteristics and MRI parameters in *CM0-SM* and *CM1-SM* groups

| Clinical characteristics | MRI parameters in groups: | | | |
| --- | --- | --- | --- | --- |
|  | *CM0-SM* | | *CM1-SM* | |
|  | positive | negative | positive | negative |
| Age of clinical manifestation | S csf iot, S csf/S fm iot  h so, trd FM sot | TTw, Z, h eb |  | T midsag  apsc С1, BA |
| Paresis | AxS4, BoA, ROST  Syrinx length  Sagittal taper PCF | Circumscribed type Sag apscord Smax  S sc Smax, X, FVSD  Ax apscord/Ax apsc  Ax apd Smax, h  AxS2, CAAm, CLgr  S tt sot, S fm sot | Syrinx length Slender type BoA | AxS2  h ant  CLgr |
| Segmental-dissociated sensory loss, % | ChD | S csf iot  S sc Smax | FM  C-PCF | Ax trd Smax  S syr max  S syr max/S vc max  S sc Smax |
| CM1-associated headache (%), including: | S tt iot  Circumscribed type | S csf/S fm iot | Distended type |  |
| - cough headache, % | SmCL pf | SC-ISCC  Slender type | Distended type, AXs2  trd FM iot  S m sot | Slender type |
| No headache, % | S pcf b, S fm sot  apd FM iot, VER-ISCC  VH index, S tt sot |  |  | S tt iot/S fm iot  TTw  Distended type |
| CSI, mean | CL, Y, AxS2, S vc max  Ax trd Smax, S syr max |  | AxS3 | h so |
| CSI 1, % | Circumscribed type | Slender type | Distended type |  |
| CSI 2, % | S tt sot  S tt sot/S fm sot  Slender type  AxS1 | S tt iot  S tt iot/S fm iot, S csf/S fm sot  S csf sot, S syr max  Vaquero Index  Circumscribed type | obex-McR | T cor, S sc Smax  Distended type Vaquero Index  Sag apd Smax  S syr max, AxS4  S syr max/S vc max Sag apscord Smax  Ax apscord  Ax apd Smax  Ax trd Smax |
| CSI 3, % | Y, CL, AxS2, S vc Smax  Ax trd Smax, S syr max  S syr max/S vc max |  | Ax trd Smax AxS3 |  |

Suppl. Table II. Positive and negative correlations (*p*<0.05) between MRI parameters in *CM0-SM* and *CM1-SM* groups

| MRI variables | MRI parameters in groups: | | | |
| --- | --- | --- | --- | --- |
|  | *CM0-SM* | | *CM1-SM* | |
|  | positive | negative | positive | negative |
| **T cor** | S tt iot, S tt/S fm iot,  S tt sot | *S csf/S fm* iot | S tt iot, S tt/S fm iot, S tt sot, S tt/S fm sot | obex-McR, S csf iot, *S csf/S fm* iot, S csf sot, *S csf/S fm* sot |
| **obex-McR** | P-FM, F-FM, h ant,  **Syrinx size:**  Sag apscord Smax /Sag apsc Smax, FVSD, AxS1,  Ax apscord/Ax apsc | apsc С1, EI, HW, S tt iot, S tt/S fm iot  **Syrinx size:**  Syrinx length | M-FM, T cor, ROST | up PCF  **Syrinx size:**  S syr max/S vc max, Vaquero Index,  Sag apd Smax,  Sag apd Smax /Sag apscord Smax,  Ax apd Smax |
| **h ant** | P-FM, C-FM, M-FM, BS-PCF, obex-McR, S pcf, S pcf b, Z, TA, CL gr, h, SmSO pcf, FVSD, CAAm, FV, CSF-PCF, h/H, rostral level of the SM cavity | BoA, BA, C-SO, sagittal taper PCF, frontal taper PCF, VHs, ChD, v, distended type | apd FM iot, S fm iot, S tt iot, P-FM, C-FM, M-FM, S pcf, S pcf b, Z, CL gr, h, h/H, SmSO pcf, TA, VER-ISCC, VH index, trd FM iot | BoA, BA, C-SO, sagittal taper PCF, frontal taper PCF,  GlSm pcf, Y, |

Suppl. Table III. Correlation between Evans index and TH

|  | *CM0-SM* | | | | *CM1-SM* | | | |
| --- | --- | --- | --- | --- | --- | --- | --- | --- |
|  | T max | | T midsag | | T max | | T midsag | |
|  | r | p | r | p | r | p | r | P |
| EI | .055 | .778 | .097 | .618 | -.116 | .452 | -.101 | .515 |

Suppl. Table IV. Correlations between parameters of the reduction, crowdedness, and flattening of the PCF with the grade of tonsils and the obex descent in *Primary CM1-SM* patients

|  | T cor | | T midsag | | obex-McR | | VH index | | VH d | | VH s | |
| --- | --- | --- | --- | --- | --- | --- | --- | --- | --- | --- | --- | --- |
|  | r | p | r | p | r | p | r | p | r | p | r | p |
| **Size of the PCF** | | | | | | | | | | | | |
| h | -.111 | .463 | -.112 | .457 | .254 | .089 | -.066 | .669 | -.079 | .612 | -.115 | .456 |
| h/H | -.150 | .331 | -.172 | .264 | .254 | .096 | -.048 | .757 | -.065 | .676 | -.113 | .465 |
| h ant. | .021 | .892 | -.042 | .783 | -.019 | .902 | .302 | .046* | .260 | .088 | .268 | .079 |
| CL | -.041 | .785 | -.135 | .373 | .127 | .401 | .273 | .073 | .220 | .151 | .295 | .051 |
| SO | -.163 | .280 | -.169 | .262 | .154 | .307 | -.328 | .030* | -.239 | .118 | -.329 | .029* |
| Exoc dex | -.011 | .958 | .158 | .440 | .079 | .383 | -.191 | .362 | -.236 | .257 | -.158 | .450 |
| Exoc sin | .098 | .635 | .204 | .317 | .118 | .567 | -.185 | .376 | -.262 | .205 | -.186 | .374 |
| V pcf | .092 | .543 | -.054 | .723 | .014 | .926 | -.162 | .294 | -.023 | .880 | -.074 | .633 |
| S pcf | -.118 | .435 | -.232 | .120 | .135 | .372 | -.270 | .077 | -.151 | .329 | -.211 | .170 |
| S pcf b | -.070 | .643 | -.160 | .287 | .082 | .589 | -.058 | .710 | -.055 | .725 | -.045 | .771 |
| S pcf eb | -.029 | .847 | -.122 | .421 | .070 | .645 | -.358 | .017* | -.191 | .214 | -.278 | .068 |
| **PCF liquor spaces and PCF crowdedness** | | | | | | | | | | | | |
| PCF CI | .264 | .083 | .253 | .098 | -.191 | .215 | -.102 | .509 | -.322 | .033* | -.102 | .511 |
| **Shape of the PCF** | | | | | | | | | | | | |
| BoA | -.010 | .949 | .000 | .999 | .244 | .102 | -.405 | .006* | -.552 | .000* | -.351 | .019* |
| ВА | .057 | .715 | .095 | .540 | .003 | .986 | -.094 | .544 | -.119 | .441 | -.116 | .455 |
| C-SO | .027 | .857 | .049 | .748 | -.140 | .355 | -.168 | .275 | -.156 | .313 | -.077 | .617 |
| TA | -.008 | .957 | -.063 | .675 | .239 | .110 | .113 | .464 | .120 | .436 | .061 | .696 |
| Sagittal taper PCF | -.017 | .912 | -.023 | .877 | .024 | .873 | -.267 | .080 | -.196 | .203 | -.175 | .257 |
| Coronal taper PCF | .207 | .172 | .206 | .175 | -.188 | .215 | -.143 | .354 | -.080 | .605 | -.098 | .526 |

* p<0.05

Suppl. Table V. Correlations between parameters of the reduction, crowdedness and flattening of the PCF with the grade of tonsils and the obex descent in *Primary CM0-SM* patients

|  | T cor | | T midsag | | obex-McR | | VH index | | VH d | | VH s | |
| --- | --- | --- | --- | --- | --- | --- | --- | --- | --- | --- | --- | --- |
|  | r | p | r | p | r | p | r | p | r | p | r | p |
| **Size of the PCF** | | | | | | | | | | | | |
| h | .254 | .130 | -.162 | .338 | .221 | .189 | -.126 | .491 | .205 | .261 | -.281 | .119 |
| h/H | .199 | .292 | -.206 | .275 | .043 | .820 | .019 | .922 | .323 | .087 | -.161 | .405 |
| h ant. | -.003 | .984 | -.278 | .095 | .369 | .024* | -.195 | .285 | .323 | .071 | -.513 | .003* |
| CL | .171 | .310 | .063 | .712 | .228 | .175 | -.177 | .332 | .250 | .167 | -.454 | .009* |
| SO | .000 | .999 | -.165 | .330 | .234 | .163 | -.084 | .647 | 0.000 | 1.000 | -.087 | .635 |
| Exoc dex | -.113 | .655 | -.281 | .259 | .213 | .397 | .216 | .405 | .293 | .253 | .013 | .959 |
| Exoc sin | -.055 | .827 | -.344 | .163 | .172 | .495 | .319 | .212 | .438 | .079 | .083 | .752 |
| V pcf | .325^*^ | .050 | .080 | .636 | .013 | .937 | -.175 | .338 | .113 | .538 | -.317 | .077 |
| S pcf | .146 | .390 | -.196 | .244 | .199 | .238 | -.229 | .207 | .260 | .150 | -.458 | .008* |
| S pcf b | .037 | .828 | -.246 | .141 | .232 | .168 | -.171 | .350 | .171 | .350 | -.368 | .038* |
| S pcf eb | .159 | .346 | -.100 | .555 | .020 | .904 | .014 | .939 | .286 | .113 | -.131 | .475 |
| **PCF liquor spaces and PCF crowdedness** | | | | | | | | | | | | |
| PCF CI | .093 | .606 | .429 | .013* | .087 | .632 | .031 | .871 | -.397^*^ | .027 | .275 | .134 |
| **Shape of the PCF** | | | | | | | | | | | | |
| BoA | -.027 | .874 | .015 | .929 | .018 | .915 | .149 | .416 | .113 | .537 | .196 | .283 |
| ВА | .089 | .628 | .204 | .262 | -.154 | .402 | .028 | .882 | -.034 | .858 | .133 | .482 |
| C-SO | -.013 | .937 | .255 | .128 | -.188 | .266 | .124 | .497 | -.061 | .738 | .146 | .426 |
| TA | .224 | .182 | -.036 | .831 | .021 | .902 | .029 | .875 | .140 | .445 | -.063 | .733 |
| Sagittal taper PCF | -.168 | .328 | .176 | .303 | -.161 | .348 | .183 | .323 | -.097 | .605 | .285 | .120 |
| Coronal taper PCF | -.275 | .128 | .190 | .298 | -.112 | .543 | -.086 | .650 | -.357 | .053 | .146 | .443 |

* p<0.05

Suppl. Table VI. Correlations between Indicators of the descent inside the PCF and parameters of the PCF reduction and flattening in *Primary CM1-SM* patients

|  | P-FM | | C-FM | | F-FM | | M-FM | |
| --- | --- | --- | --- | --- | --- | --- | --- | --- |
|  | r | p | r | p | r | p | r | p |
| CL | .596 | .000* | .536 | .000* | .245 | .100 | .423 | .003* |
| V pcf | .089 | .554 | .320 | .032* | .158 | .295 | .150 | .320 |
| S pcf | .287 | .053 | .554 | .000* | .254 | .088 | .317 | .032* |
| S pcf b | .297 | .045* | .462 | .001* | .137 | .364 | .326 | .027* |
| BoA | -.405 | .005* | -.374 | .011* | -.072 | .636 | -.010 | .945 |
| C-SO | -.317 | .032* | -.364 | .014* | -.056 | .711 | -.254 | .088 |

* p<0.05

Suppl. Table VII. Correlations between Indicators of the descent inside the PCF and parameters of the PCF reduction and flattening in *Primary CM0-SM* patients

|  | P-FM | | C-FM | | F-FM | | M-FM | |
| --- | --- | --- | --- | --- | --- | --- | --- | --- |
|  | r | p | r | p | r | p | r | p |
| CL | .532 | .001* | .456 | .008* | .058 | .736 | .311 | .061 |
| V pcf | .336 | .049* | .274 | .122 | .169 | .324 | .217 | .197 |
| S pcf | .565 | .000* | .468 | .006* | .470 | .004* | .421 | .009* |
| S pcf b | .572 | .000* | .485 | .004* | .303 | .072 | .390 | .017* |
| BoA | -.449 | .007* | -.348 | .047* | .002 | .989 | -.103 | .545 |
| C-SO | -.323 | .059 | -.463 | .007* | -.268 | .114 | -.145 | .392 |

* p<0.05

Suppl. Table VIII. Correlations between indicators of the PCF volume and size with parameters of osseous dimension of FM

|  | *CM0-SM* | | | | *CM1-SM* | | | |
| --- | --- | --- | --- | --- | --- | --- | --- | --- |
|  | V pcf | | S pcf | | V pcf | | S pcf | |
|  | r | p | r | p | r | p | r | p |
| apd FM iot | .205 | .223 | .087 | .607 | .044 | .774 | .090 | .554 |
| S fm iot | .280 | .114 | .180 | .316 | .031 | .838 | .055 | .720 |
| S csf/S fm iot | -.317 | .077 | -.214 | .239 | .163 | .291 | .095 | .538 |
| S m iot | .459 | .008* | .373 | .035* | -.027 | .860 | .054 | .728 |
| BS-PCF | .552 | .001* | .602 | .000* | .368 | .014* | .397 | .008* |

* p<0.05

Suppl. Table IX. Correlations between Indicators of the PCF entity and parameters of the FM

|  | *CM0-SM* | | | | *CM1-SM* | | | |
| --- | --- | --- | --- | --- | --- | --- | --- | --- |
|  | S pcf b |  | CL |  | S pcf b |  | CL |  |
|  | r | p | r | p | r | p | r | p |
| apd FM iot | .168 | .319 | .137 | .418 | .346 | .018* | .317 | .032* |
| S fm iot | .256 | .151 | .201 | .261 | .368 | .013* | .391 | .008* |
| apd FM sot | .421 | .009* | .282 | .091 | -.155 | .304 | -.102 | .501 |
| S fm sot | .462 | .007* | .268 | .131 | -.041 | .791 | .017 | .912 |

* p<0.05

Suppl. Table X. Correlations between angular Indicators of the PCF and parameters of the FM in *Primary CM1-SM* patients

|  | TTw | | TA | | BoA | | C-SO | |
| --- | --- | --- | --- | --- | --- | --- | --- | --- |
|  | r | p | r | p | r | p | r | p |
| S fm iot | -.128 | .403 | .298 | .047* | -.325 | .029* | -.356 | .017* |
| S csf/S fm iot | -.217 | .157 | -.336 | .026* | .110 | .477 | .364 | .015* |
| S tt/S fm iot | .209 | .174 | .368 | .014* | -.187 | .225 | -.400 | .007* |

* p<0.05

Suppl. Table XI. Correlations between angular Indicators of the PCF and parameters of the FM in *Primary CM0-SM* patients

|  | TTw | | TA | | BoA | | C-SO | |
| --- | --- | --- | --- | --- | --- | --- | --- | --- |
|  | r | p | r | p | r | p | r | p |
| S fm iot | .142 | .429 | .337 | .055 | -.460 | .007* | -.301 | .089 |
| S csf/S fm iot | -.392 | .027* | -.356 | .045* | -.136 | .460 | .049 | .789 |
| S tt/S fm iot | .383 | .030* | .445 | .011* | -.069 | .708 | -.146 | .424 |

* p<0.05

Suppl. Table XII. Correlations between parameters of the syrinx size and the grade of TH and the FM crowdedness

| variables | Ax trd Smax | | S syr max | | Sag apd Smax | | Sag apd syr / Sag apscord | | Vaquero Index | | Ax apd Smax | |
| --- | --- | --- | --- | --- | --- | --- | --- | --- | --- | --- | --- | --- |
|  | r | p | r | p | r | p | r | p | r | p | r | p |
| *CM0-SM* | | | | | | | | | | | | |
| T max | .174 | .317 | .116 | .506 | .166 | .327 | .160 | .345 | .132 | .435 | .158 | .351 |
| S tt iot | .235 | .195 | .154 | .401 | .131 | .476 | .136 | .458 | .105 | .568 | .147 | .421 |
| S csf/S fm iot | -.202 | .267 | -.087 | .634 | .084 | .647 | .083 | .653 | .116 | .527 | .014 | .940 |
| S tt sot | -.051 | .783 | .096 | .601 | -.271 | .134 | -.261 | .149 | -.245 | .176 | .091 | .620 |
| S csf/S fm sot | .291 | .106 | .149 | .415 | .334 | .062 | .266 | .142 | .321 | .074 | .011 | .952 |
| T midsag | .033 | .852 | -.033 | .853 | -.239 | .154 | -.135 | .426 | -.232 | .167 | -.130 | .443 |
| *CM1-SM* | | | | | | | | | | | | |
| T max | .222 | .138 | .251 | .093 | .195 | .194 | .189 | .207 | .230 | .125 | .252 | .091 |
| S tt iot | .143 | .355 | .218 | .154 | .169 | .273 | .147 | .339 | .182 | .237 | .198 | .196 |
| S csf/S fm iot | -.159 | .303 | -.237 | .121 | -.234 | .126 | -.242 | .114 | -.251 | .100 | -.188 | .222 |
| S tt sot | .083 | .590 | .061 | .694 | -.038 | .804 | -.065 | .675 | -.042 | .789 | -.002 | .991 |
| S csf/S fm sot | -.143 | .353 | -.120 | .436 | -.005 | .974 | .007 | .966 | -.023 | .883 | -.051 | .743 |
| T midsag | .120 | .427 | .199 | .185 | .154 | .307 | .155 | .304 | .199 | .185 | .219 | .143 |
